# Supplementary material for: Chromatin 3D structure reconstruction with consideration of adjacency relationship among genomic loci
Source: BMC Bioinformatics. 2020 Jul 1;21:272. doi: 10.1186/s12859-020-03612-4 (PMC7329537; doi:10.1186/s12859-020-03612-4)
Supplement: Supplementary file 1 — Additional file 1. [file 12859_2020_3612_MOESM1_ESM.docx]

chromatin 3D structure reconstruction with consideration of adjacency relationship among genomic loci

Supplementary Materials

**1 Proof of Eq.(7)**

We firstly convert the second term of Eq.(6) into matrix forms by the following derivation:

$\begin{aligned} \sum_{i,j} m_{ij}\left\| x_{i}-x_{j} \right\|^{2} \\ \text{=}\sum_{i,j} m_{ij}\left( x_{i}-x_{j} \right)'\left( x_{i}-x_{j} \right) \\ \text{=}2\sum_{i,j} m_{ij}x_{i}^{'}x_{i}-2\sum_{i,j} m_{ij}x_{i}^{'}x_{j} \\ \text{=}2\sum_{i} d_{ii}x_{i}^{'}x_{i}-2\sum_{i,j} m_{ij}d_{ii}x_{i}^{'}x_{j} \\ \text{=}2tr\left( X^{'}DX \right)-2tr\left( X^{'}MX \right) \\ \text{=}2tr\left( X'LX \right) \end{aligned}$ (S1)

The above derivation made use of the symmetry property of the affinity matrix *M*. Remember $L=D-M$ is Laplacian matrix and D the diagonal matrix with entries$d_{ii}=\sum_{j} m_{ij}$. Then Eq.(7) becomes

$\psi=tr\left( B-\hat{B} \right)^{2}+2\rho tr\left( X'LX \right)=tr\left( B-HXX'H \right)^{2}+2\rho tr\left( X'LX \right)$ (S2)

Note that *L* satisfies $L=LH=HL$. Substituting the centralized coordinates $Y=HX$ into (S2) we get

$\begin{aligned} \psi=tr\left( B-YY' \right)^{2}+2\rho tr\left( Y'LY \right) \\ \text{=}tr\left( B^{2}-BYY'-YY'B+YY'YY'+2\rho Y'LY \right) \\ \text{=}tr\left( B^{2}-2BYY'+YY'YY'+2\rho LYY' \right) \\ \text{=}tr\left[ YY'-\left( B-\rho L \right) \right]^{2}+tr\left[ B^{2}-\left( B-\rho L \right)^{2} \right] \end{aligned}$ (S3)

The second trace of (S3) has nothing to do with the coordinates matrix *X* to be solved and so can be removed from the object function. Hence, by denoting $\tilde{B}=\left( \tilde{b_{ij}} \right)=B-\alpha L$ and remembering $\hat{B}=HXX'H=YY'$, we obtain the final object function to be minimized as

$\tilde{\psi}=tr\left[ YY'-\left( B-\rho L \right) \right]^{2}=tr\left[ \left( B-\rho L \right)-YY' \right]^{2}=tr\left( \tilde{B}-\hat{B} \right)^{2}$ (S4)

**2 Supplementary figures**

**Figure S1. RMSD difference, i.e. RMSD of ShRec3D, ShNeigh1 and ShNeigh2 minus that of ChromSDE on simulated data under varying number of nearest neighbors *K*.**

**Figure S2. Estimated conversion factor of GM06990 Hi-C data at different resolutions for (a) HindIII enzyme and (b) NcoI enzyme.** Averaged across 23 chromosomes.

**Figure S3. Performance of different methods on GM06990 Hi-C data at different resolutions for each chromosome.** The left column corresponds to RMSD measure and the right column corresponds to dSCC measure, and each row represents one resolution.
